# New Technique for Automatic Segmentation of Blood Vessels in CT Scan Images of Liver Based on Optimized Fuzzy *C*-Means Method

**DOI:** 10.1155/2016/5237191

**Published:** 2016-12-04

**Authors:** Katayoon Ahmadi, Abbas Karimi, Babak Fouladi Nia

**Affiliations:** Department of Computer Engineering, Faculty of Engineering, Arak Branch, Islamic Azad University, ARAK, Markazi, Iran

## Abstract

Automatic segmentation of medical CT scan images is one of the most challenging fields in digital image processing. The goal of this paper is to discuss the automatic segmentation of CT scan images to detect and separate vessels in the liver. The segmentation of liver vessels is very important in the liver surgery planning and identifying the structure of vessels and their relationship to tumors. Fuzzy *C*-means (FCM) method has already been proposed for segmentation of liver vessels. Due to classical optimization process, this method suffers lack of sensitivity to the initial values of class centers and segmentation of local minima. In this article, a method based on FCM in conjunction with genetic algorithms (GA) is applied for segmentation of liver's blood vessels. This method was simulated and validated using 20 CT scan images of the liver. The results showed that the accuracy, sensitivity, specificity, and CPU time of new method in comparison with FCM algorithm reaching up to 91%, 83.62, 94.11%, and 27.17 were achieved, respectively. Moreover, selection of optimal and robust parameters in the initial step led to rapid convergence of the proposed method. The outcome of this research assists medical teams in estimating disease progress and selecting proper treatments.

## 1. Introduction

Liver is a vital organ in the body that cleans the body from toxins and harmful substances. Due to the function of liver, it is vulnerable to cancer. Resection and transplant of the tumor are two main treatments in routine clinical practice while both need planning and quality assessment of CT images for further processing.

Conventionally, detection of malignant tumors in CT scan images is visually applied by experienced physicians but it is time consuming and subject to human errors. Furthermore, CT scan images usually have low contrast and low visibility which increase the probability of false positive and false negative in cancer detection by human observers.

Comprehensive understanding of vascular anatomy of the liver is vital to cut segment-circuit which is derived from Couinaud's descriptions about the liver segmentation. It is based on distribution of the venous shoots and the position of the liver vessels [1]. Liver surgery based on computer supported planning has a great effect on therapeutic strategies. An important step in the preoperation phase is to visualize the relationship between tumor, liver, and vascular tree of each patient [2].

## 2. Related Works

So far, multiple image processing techniques have been introduced in the literature for automatic segmentation of liver vessels. Threshold method is perhaps the most important segmentation technique in medical image processing. Selection of proper threshold is the common problem of all threshold-based techniques. In [3], a three-level thresholding method by using fuzzy entropy and genetic algorithm (GA) was presented that degrades problem of threshold selection. Fuzzy *C*-means (FCM) in conjunction with GA was discussed in [4] where the results showed a significant improvement in the accuracy of image segmentation in comparison with other methods. Another study in [5] introduced a modified FCM technique by a regularizing functional and a regularization parameter to balance clustering and smoothing in noisy and incomplete images.

In [6], a region growing vessel segmentation algorithm based on spectrum information was introduced which applied Fourier transform on the Region of Interest (ROI). ROI included vascular structures to gain spectrum information according to extracted primary features. Then combined edge information with primary feature direction computed the center points of the vascular structure as the seeds of region growing segmentation. Finally, improved region growing method with branch-based growth strategy is used to segment the vessels. In order to measure the effectiveness of this algorithm, the results on retinal and abdomen liver vascular CT scan images examined and showed that the proposed method can not only extract the high quality target vessel region, but also reduce effectively the manual intervention.

A novel 4D graph-based method to segment hepatic vasculature and tumors was introduced in [7]. The algorithm uses multiphase CT images to model the differential enhancement of the liver structures and Hessian-based shape likelihoods to avoid the common pitfalls of graph cuts under segmentation and intensity heterogeneity. Veins were tracked using the graph representation and planes fitted to the vessel segments. The method allows the detection of all hepatic tumors and identification of the liver segments with 87.8% accuracy.

Multiple automatic segmentations of liver vessels have been taken into account, for example, adaptive threshold [8], region growing [9], fuzzy *c*-mean clustering, and level set [10]. Adaptive threshold method [8] utilized a two-phased image segmentation including conversion of gray scale to binary image and adapting the threshold value by applying a generated binary mask on the CT scan image. Region growing [9] is performed in the first and the third phases of CT scan by tracing the portal vein and the hepatic vein. Tracing the veins by a 3D labeling operation in the ROI is performed using a threshold. Threshold value helps in separating the blood vessels from the liver's soft tissue. Erosion and dilation operations remove the adjoining stomach and spleen regions. Morphological dilation and an optimal threshold value are applied to estimate the region of the liver. The analysis showed comparable results of this method in comparison with manual detection of the regions by an expert. In [10], spatial fuzzy *c*-means clustering combined with anatomical prior knowledge is employed to extract liver region, while a distance regularized level set is used for refinement followed by morphological operations. The experiment result shows a high accuracy (0.9986) and specificity (0.9989).

Fast FCM (FFCM) was optimized by Particle Swarm Optimization (PSO) [1] and applied on liver CT images. Evaluation performance was performed in terms of Jaccard Index and Dice Coefficient and based on ANOVA analysis showing higher values than FFCM.

High sensitivity to noisy data and being trapped in local optimum are common disadvantages of FCM and other methods as already mentioned. As the idea of using image energy [11] resolved these problems, in this study, a combination of FCM and GA is presented. Energy-based approach [11] achieved an initial segmentation closed to the liver's boundary followed by an Active Contour Model (ACM) and GA to produce a proper parameter set closed to the optimal solution. This method had a better ability to segment the liver tissue with respect to the other methods. Furthermore, it showed high accuracy, precision, sensitivity, specificity and low overlap error, MSD, and runtime with fewer ACM iterations.

In this paper, an Innovative heuristic method is introduced based on FCM and GA. By using FCM and GA and selecting suitable features for the liver segmentation in CT images, the sensitivity to noisy data and the heavy dependency on initial data are degraded.

## 3. Fuzzy Clustering

Fuzzy clustering can be considered as a part of fuzzy data analysis and has two parts: fuzzy data analysis and deterministic data analysis using fuzzy techniques. In the latter, each cluster is assumed as a set of elements. Then by changing the definition of membership in which “an element can only be a member of a cluster (the partition mode)” to the definition that “any element can be a member of multiple clusters with different membership degrees,” classification becomes more compatible with reality [12].

### 3.1. Fuzzy *C*-Means Algorithm

Fuzzy *C*-means method is one of the most common methods involving feature analysis, clustering, and classifier design. Like the *K*-Means method, FCM is a family member of the clustering algorithms with an objective function where all seek to minimize the function. Using fuzzy membership, FCM algorithm identifies the pixels corresponding to each group.

The first version of FCM algorithm was presented by Duda and Hart (1973) to perform an accurate clustering. Since some data were dependent on multiple clusters, it was impossible to merge them into a single cluster. This algorithm was revised several times. Finally, Bezdek proposed its final version by defining *m* as fuzzifier [13]. The resulting algorithm identifies spherical clouds of points in a P-dimensional space. These clusters are supposed to have roughly the same size. Each cluster is displayed with a centroid. In selecting the centroid, the mean value is used as a representative of all data assigned to the cluster. To calculate the centroid, total degrees of membership of each element are divided by the product of degrees of membership raised to the power *m*. This algorithm does not recognize clusters in different shapes, sizes, and densities. In practice, the final solution can be reached with a few iterations. Therefore, this algorithm reduces the computational time. In other types of FCM, rather than using the identity matrix in determining the distance, matrices such as diagonal might be used for segmentation of the elliptic clusters.

Assume that *x*_*j*_  (*j* = 1,2,…, *n*) represents an image with *n* pixels separated in *C* classes, where *x*_*j*_ represents data for shapes. The ISAN classic iterative optimization algorithm that reduces the cost function is defined as shown below.

(1) J=∑i=1c∑j=1nuijmdij2dij=ci−xj,

where *u*_*ij*_ represents the membership of *x*_*j*_ in the *i*th cluster, *u*_*ij*_ ∈ [0,1]. *c*_1_ is the center of the *i*th class and *m* is a constant. Parameter *m* controls the fuzziness of the segment. The value of cost function is directly related to the Euclidean distances between the center and pixels, that is, nearness of pixels to the center reduces the cost function and increases the membership values, while pixels far from the center have low member ship values in higher costs. The membership function indicates the probability that a pixel belongs to a specified class. In the FCM algorithm, this probability only depends on the distance between the pixel and the center of each separate class in a specified area. Membership functions and class centers are modified and updated by the following formula:

(2) uij=1∑k=1cdij/dkj2/m−1ci=∑j=1nuijmxj∑j=1nuijm.

Standard FCM algorithm is called optimized when the values of the pixels are high near the center and have low algebra values in locations far from the center. One of the disadvantages of the standard FCM used for the image separation and segmentation is that it only uses gray-level intensity information with no use of spatial data of pixels. In fact, the probability that gray-level intensity of adjacent pixels belonged to the same class is high.

Chuang et al. proposed a spatial FCM algorithm which merges spatial data with fuzzy membership functions. The spatial function is defined as follows.

(3) $$\begin{array}{c} h_{ij}=\underset{k\in NB\left(x_{j}\right)}{\sum }u_{ik}, \end{array}$$

where NB(*x*_*j*_) represents a square window centered on pixel *x*_*j*_ in the spatial field. Similar to membership function, the *h*_*ij*_ spatial function represents the probability that the majority of neighboring pixels belong to the same class. The spatial function is combined with the membership function as follows.

(4) $$\begin{array}{c} u_{ij}^{\prime }=\frac{u_{ij}^{p}h_{ij}^{q}}{\sum_{k=1}^{c}u_{kj}^{p}h_{kj}^{q}}, \end{array}$$

where *p* and *q* control the relative importance of *u*_*ij*_ and *h*_*ij*_[10].

## 4. Genetic Algorithm

Genetic algorithm (GA) is a well-known biological-based method taken from the genetics. The GA produces a very large set of possible solutions for a given problem. Each of these solutions is evaluated using a fitness function. Then some of the best solutions are utilized in producing new solutions as the evolved solutions. Hence, the solution is progressed until reaching an optimal one. The effectiveness of GA highly depends on proper selection of parameters [14].

Every solution for a given problem is represented in a list of parameters called chromosome. The most common method of representing chromosomes in the GA is in the form of binary strings, although other representations might be used. Initially, multiple features are randomly created as the first generation. Along with a generation, every single feature is evaluated by a fitness function and its fitness value is measured.

The next step of GA is generating the second generation according to proper selection of features in previous step. This is done by using genetic operators, for example, chromosome join and chromosome modification. Parents are selected for reproduction and are combined using crossover or mutation operators in order to produce new offspring in such a way that the best elements are chosen. Even the weakest elements have the chance of selection to avoid local answer. There are different selection methods in literature including roulette and tournament. This process is repeated until the next generation of population is produced. GA has a probability of connection between 0.6 and 1 showing the probability of generating a child. By connecting two different chromosomes a child is generated and connected to the next generation. This process continues until a good candidate (answer) is found in next generation. This process causes new generations of chromosomes which are different from previous generations. In every step, the population is studied. On the condition that the convergence criteria are met, the process is terminated. Other criteria for termination of GA are runtime and the number of generations.

## 5. The Proposed Method

Automatic detection of liver cancer based on blood vessels consists of three main steps. They are preprocessing, image segmentation, and ROI classification.

**Figure 1:**
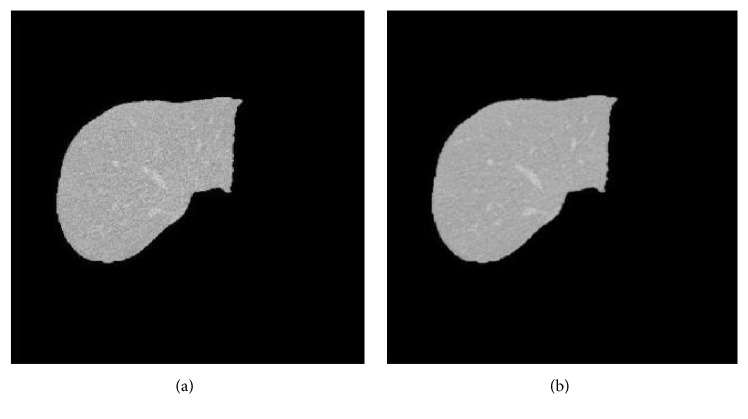
Preprocessing image on the original CT scan images. (a) Image before applying the filter and (b) image after applying the filter (preprocessing).

Main problem of the segmentation algorithms is the handling of inhomogeneous or insufficient contrasted images. In line with this, the proposed method initially enhances the quality of CT image by removing noises. This process is known as preprocessing: an input CT image is passed through a Gaussian low-pass filter with relatively large kernel, the noise is eliminated, and a sharper image is obtained.  Figure 1 shows the given original image and the image after preprocessing. It is noted that handling of partial volume effects in smaller vessels is not taken into account in this study.

Segmentation of blood vessels in the liver's CT image is performed by combination of FCM and GA. This method starts with selection of random values from the original CT image. Random values are assumed as the core centers (chromosomes) which feed FCM segmentation procedure.

The image is clustered by FCM. Membership function of GA is applied on the values of all pixels in each FCM cluster. Centroid of each cluster is then calculated considering soft values of Euclidian distances from the center point which mimics the calculation of the cost function in FCM algorithm. The values of cost function are considered as the cost value of chromosomes. These values are then used as the basis for sorting the population members.

**Figure 2:**
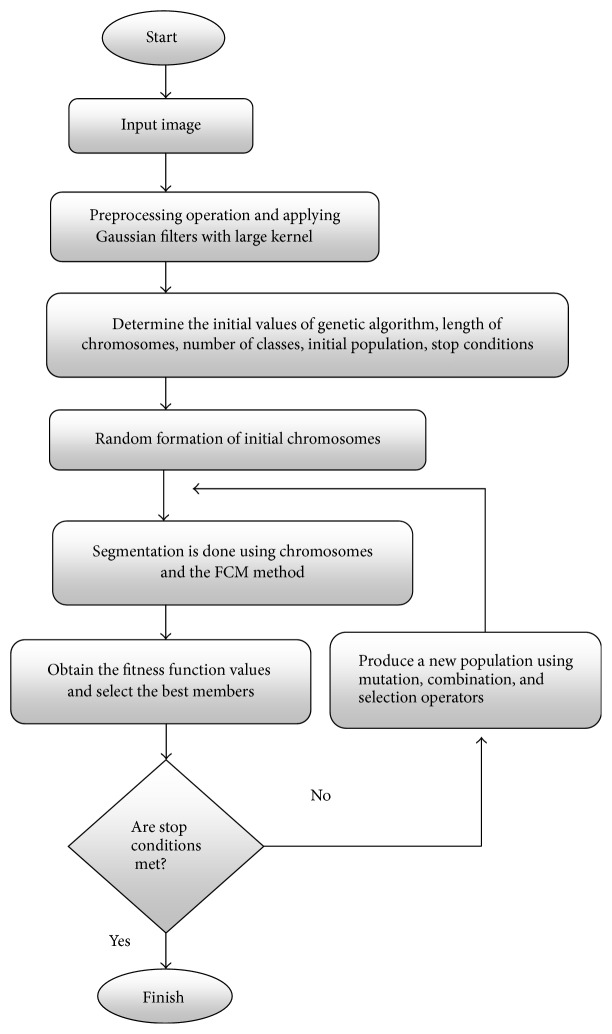
Flowchart of the proposed algorithm.

**Figure 3:**
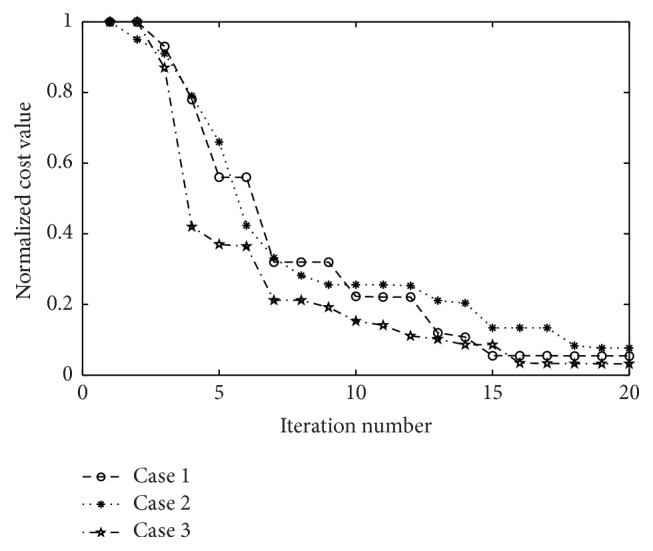
The cost amounts to three cases.

According to mutation and crossover percentages, fitness of new members of population refeeds the FCM algorithm and cost values are obtained (Figure 3). Convergence criteria is checked and algorithm continues if it is not achieved yet. General procedure of the proposed algorithm is delineated in Figure 2.

## 6. Results and Discussion

In order to validate the proposed algorithm 20 gray scale images of liver's CT scan with the size of 512*∗*512 (2D) were used. All training and test data from the presented study are publicly available on the web (see http://www.sliver07.org/). All simulations were executed using MATLAB® and on a system with Core i5, RAM 4 MB.

The number of population is considered 100, chromosome's length is 6, the number of iterations is 40, crossover is 60%, mutation is 30%, Elitistism is 10%, and the initial value for chromosomes is random between (0,255).  Table 1 shows the parameters of the proposed method for segmentation of the original CT scan images.

In Figure 5, normalized cost values for the first three to 20 repetitions are shown. As seen the cost is reduced considerably showing that algorithm converges after 20 iterations.

**Figure 4:**
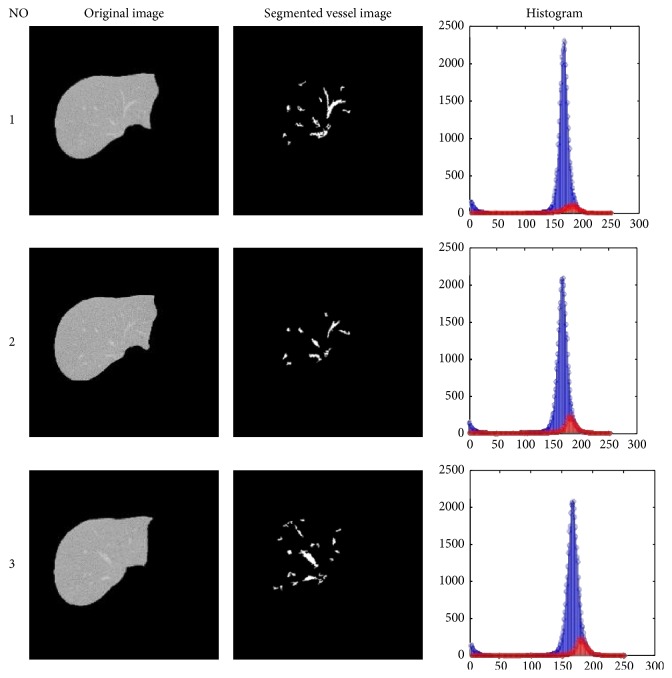
Simulation results on CT scan images of the liver. This figure shows post-pre processing images, segmented vessel images, and histogram images. In the histogram section, the blue graph presents the original image's histogram and the red graph refers to the segmented vessel images' histogram of the same image.

**Figure 5:**
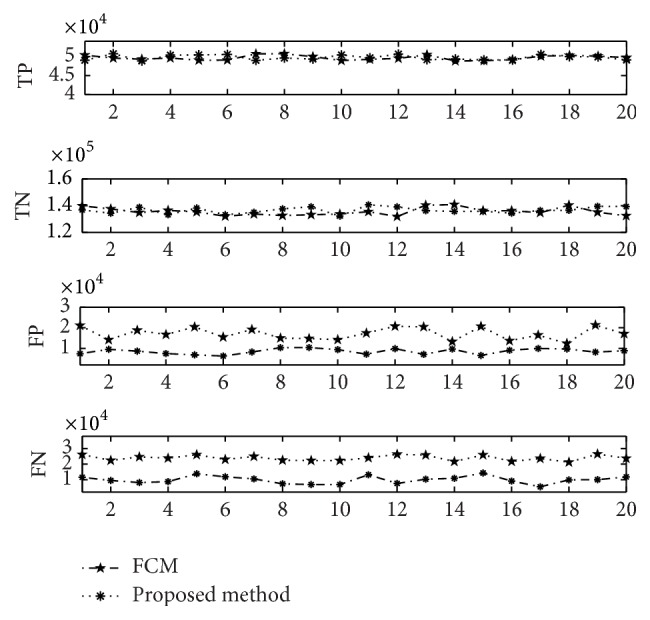
Comparison of FN, TN, FP, and TP.

The CT images of the liver are displayed in Figure 4 for three given tests, before and after execution of the proposed method. Segmented vessel image shows the spots that are reported as malignant.

Finally, the simulation values for 20 CT images were stored and corresponding rates ofaccuracy, specificity, and sensitivity have been calculated in accordance with

(5) senestivity=NTPNTP+NFNSpecificty=NTNNTN+NFPAccuracy=NTP+NTNNTP+NFN+NTN+NFP.

The calculated values of the average accuracy, specificity, runtime, and sensitivity are shown in Table 2 for the proposed method and the classical FCM method. The results showed that the proposed method overflows traditional FCM in all cases. The mean values of improvement of the proposed method in terms of accuracy, specificity, CPU time, and sensitivity were achieved as 91.01%, 94.11%, 27.17, and 83.62%, respectively.

TP as true positive, TN as true negative, FP as false positive, and FN as false negative have been calculated and presented in Table 3. As seen, the proposed method has better results compared to the standard FCM. As the results came from proper locations of the clusters (near to center) in regard to the number of categories, the decrease in FN rate and increase in FP rate occurred, which consequently increased the value of accuracy and specificity.

Values for TP, TN, FP, and FN are also compared in Figure 4. As delineated, the rates of mentioned values are better for the proposed method in companion with FCM. Therefore, the aim of this study for optimizing the FCM algorithm by using GA is achieved.

## 7. Conclusions and Future Works

This paper provided a new method based on FCM segmentation algorithm and genetic optimization algorithm for automatic segmentation of blood vessels in the CT scan images of the liver. The proper vessel segmentation in the liver images is highly desirable and greatly helps physicians during the liver surgery. The proposed method has major advantages over the classical FCM method. The simulation results showed that it achieved an accuracy of 94%, sensitivity of 83.62%, and specificity of 94.11% which are higher than FCM algorithm. Although the runtime and computational complexity of the proposed method are a little more than the FCM method, this time difference can be ignored in medical work to reach higher accuracy and sensitivity.

In future works, length of chromosomes will not be considered as default, but the length of each chromosome will be determined correspondingly in each cycle.

## Figures and Tables

**Table 1:** Parameters of genetic operations.

Parameters	Original value
Number of population	100
Chromosome's length	6
Number of iterations	40
Crossover	60%
Mutation	30%
Elitistism	10%
Initial value for chromosomes	Ran(0,255)

**Table 2:** Obtained numerical values of parameters for evaluating the methods.

	Accuracy		Specificity		CPU time (s)		Sensitivity	
	Proposed method	FCM	Proposed method	FCM	Proposed method	FCM	Proposed method	FCM
1	90.8162	79.9731	94.8538	86.8731	26.4633	13.3803	81.4028	66.0423
2	90.6447	83.7657	93.3653	90.6657	28.1738	12.9897	84.0966	69.3967
3	91.7966	80.8673	94.1179	87.7673	26.2593	12.8417	85.9281	66.0162
4	91.8856	82.1712	94.6972	89.0712	27.7998	13.0039	85.1578	67.5289
5	90.0606	79.9939	95.2727	86.8939	27.8846	12.6965	78.7255	65.8467
6	90.9693	82.6119	95.4779	89.5119	27.9874	12.7320	80.4873	68.2596
7	90.8368	80.5778	94.2416	87.4778	26.4189	13.5421	82.9291	66.2105
8	91.4389	82.9245	93.0159	89.8245	27.0496	13.5561	87.4726	68.2476
9	91.6281	83.1353	93.0479	90.0353	26.7697	13.1752	87.8618	68.6122
10	91.7641	83.4889	93.3725	90.3889	27.8501	12.6598	87.616	69.1785
11	90.3281	81.7032	95.1222	88.6032	27.1128	12.8348	79.2888	66.7889
12	91.5391	79.5029	93.3628	86.4029	28.0713	12.9532	86.8553	65.4594
13	91.4653	80.3739	95.0429	87.2739	26.6137	13.4212	82.8265	66.2031
14	89.9878	84.4800	93.3306	91.3800	26.7776	12.6154	81.8539	70.3916
15	89.8570	79.9143	95.3878	86.8143	26.5411	12.6430	77.7472	65.8231
16	90.9951	83.9549	93.6500	90.8549	26.5221	12.7690	84.6546	69.4204
17	92.3792	82.2301	93.1898	89.1301	27.9886	13.2491	90.2483	67.8824
18	90.5212	84.9768	93.3533	91.8768	27.4094	13.3317	83.6607	70.8854
19	91.2558	79.4691	94.4481	86.3691	27.3497	13.2477	83.7154	64.8601
20	90.1714	81.6561	94.0199	88.5561	26.5399	13.0509	81.2759	67.0796

*Mean*	*91.017*	*81.888*	*94.118*	*88.788*	*27.179*	*13.034*	*83.6204*	*67.5067*

**Table 3:** Parameter values of FN, TN, FP, and TP.

	TP		TN		FP		FN	
	Proposed method	FCM	Proposed method	FCM	Proposed method	FCM	Proposed method	FCM
1	50437	49685	137872	134828	7480	20977	11238	26230
2	50619	49570	131672	131166	9357	13782	9686	21769
3	49061	50338	139806	136374	8737	19007	8078	25168
4	50634	50398	140655	138306	7876	16970	8821	23974
5	50072	49181	138102	136224	6852	21150	13666	26220
6	49002	49787	138892	133908	6578	16510	12031	23627
7	49364	49699	138746	136787	8478	19581	10404	25369
8	49901	50100	135237	132701	10154	15033	7267	22609
9	50722	50226	137870	132808	10301	14699	6831	22431
10	50737	50317	133027	133190	9442	14237	7088	22193
11	49123	49359	138375	132722	7096	17972	12821	24371
12	50749	50167	131633	131158	9358	21065	7341	26379
13	50722	50118	134084	132458	6993	20335	10152	25956
14	49778	49133	131777	130750	9417	12617	11194	20981
15	50408	49045	132286	130608	6396	21356	14292	26311
16	49091	49804	139549	134815	9462	13570	9204	21714
17	49651	50727	138263	133281	10104	16254	5407	23510
18	50639	49488	134486	133826	9575	11832	9944	20576
19	50392	49978	140817	137475	8278	21697	9572	26732
20	50726	49255	131659	131048	8374	17581	11555	24047

*Mean*	*50091*	*49736*	*136240*	*13002*	*85336*	*17311*	*9829*	*24008*
